# Glycated Hemoglobin and Risk of Arterial Stiffness in a Chinese Han Population: A Longitudinal Study

**DOI:** 10.3389/fendo.2022.854875

**Published:** 2022-04-29

**Authors:** Ze Han, Xiaoping Kang, Jie Zhang, Jinqi Wang, Yue Liu, Jia Liu, Zhiyuan Wu, Xia Li, Xiaoyu Zhao, Xiuhua Guo, Shuo Chen, Lixin Tao

**Affiliations:** ^1^ School of Public Health, Capital Medical University, Beijing, China; ^2^ Department of Epidemiology and Health Statistics, Beijing Municipal Key Laboratory of Clinical Epidemiology, Beijing, China; ^3^ Rehabilitation Centre, Beijing Xiaotangshan Hospital, Beijing, China; ^4^ Department of Public Health, School of Medical and Health Sciences, Edith Cowan University, Perth, WA, Australia; ^5^ Department of Mathematics and Statistics, La Trobe University, Melbourne, VIC, Australia; ^6^ Information Department, Beijing Physical Examination Center, Beijing, China

**Keywords:** glycated hemoglobin, arterial stiffness, longitudinal study, brachial-ankle pulse wave velocity, ankle brachial index

## Abstract

**Background and Aims:**

Glycated hemoglobin (HbA1c) associates with the risk of arterial stiffness, and such association can be found between fasting blood glucose (FBG), postprandial blood glucose (PBG), triglyceride-glucose index (TyG index), and arterial stiffness. However, the results were inconsistent, longitudinal studies were sparse, and comparison of these glycemic parameters was less conducted. We aimed to explore the longitudinal relationship between HbA1c and arterial stiffness and compare the effect of the parameters.

**Methods:**

Data were collected from 2011 to 2019 in Beijing Health Management Cohort (BHMC) study. Cox proportional hazard models were fitted to investigate the association between the parameters and arterial stiffness. A generalized estimation equation (GEE) analysis was conducted to investigate the effect of repeated measurements of glycemic parameters. A receiver operating characteristic (ROC) analysis was performed to compare the predictive value of glycemic parameters for arterial stiffness.

**Results:**

Among 3,048 subjects, 591 were diagnosed as arterial stiffness during the follow-up. The adjusted hazard ratio (HR) [95% confidence interval (CI)] for arterial stiffness of the highest quartile group of HbA1c was 1.63 (1.22–2.18), which was higher than those of FBG, PBG, and TyG index. The nonlinear association of arterial stiffness with HbA1c and PBG was proved. The robust results of the sensitivity analysis were obtained.

**Conclusions:**

HbA1c is an important risk factor of arterial stiffness compared with PBG, FBG, and TyG index, and has a strong predictive ability for arterial stiffness among non-diabetics and the general population.

## Introduction

Arterial stiffness, an important pre-stage status of disease, has a dramatic effect on the progression of severe vascular diseases (1). Many studies have investigated the risk factors of arterial stiffness (2). As one of the products in the process of long-term adverse glycation, glycated hemoglobin (HbA1c) would describe the risk of not only arterial stiffness, but also cardiovascular diseases (3). Many indicators diagnosing arterial stiffness are adopted (4–7). Brachial-ankle pulse wave velocity (baPWV) and ankle brachial index (ABI) are two effective methods for the definition of arterial stiffness. Many studies have demonstrated the effect (6–9) and discriminatory power (10) of HbA1c on arterial stiffness. However, in some other studies, such association did not exist (5, 11). There exists interaction between arterial stiffness and diabetes mellitus. Recently, many studies have investigated the relationship between glucose parameters and arterial stiffness (12–14), such as fasting blood glucose (FBG) and postprandial blood glucose (PBG). Compared with FBG and PBG, HbA1c is a predictive indicator of arterial stiffness among non-diabetics (15, 16). However, the results were inconsistent (12, 15, 17). Triglyceride-glucose index (TyG index) is an indicator of insulin resistance, and is closely associated with the progression of arterial stiffness and atherosclerosis (18, 19). However, to the best of our knowledge, there is no study about the comparison between these parameters with the risk of arterial stiffness.

Currently, many studies have explored the relationship between HbA1c and risk of arterial stiffness, but studies comparing the effect of HbA1c, FBG, PBG, and TyG index on arterial stiffness were sparse. Additionally, most of the studies were cross-sectional (15, 16), and the results were inconsistent (4, 20, 21). Repeated measurement of glucose parameters was not fully considered, and the predictive ability of these parameters for arterial stiffness was not clarified and compared.

In this cohort study, we aimed to explore the longitudinal relationship between HbA1c and the risk of arterial stiffness; compare the effect of HbA1c, PBG, FBG, and TyG index on arterial stiffness; and provide effective information for population with different levels of the parameters.

## Materials and Methods

### Study Population

**Figure 1:**
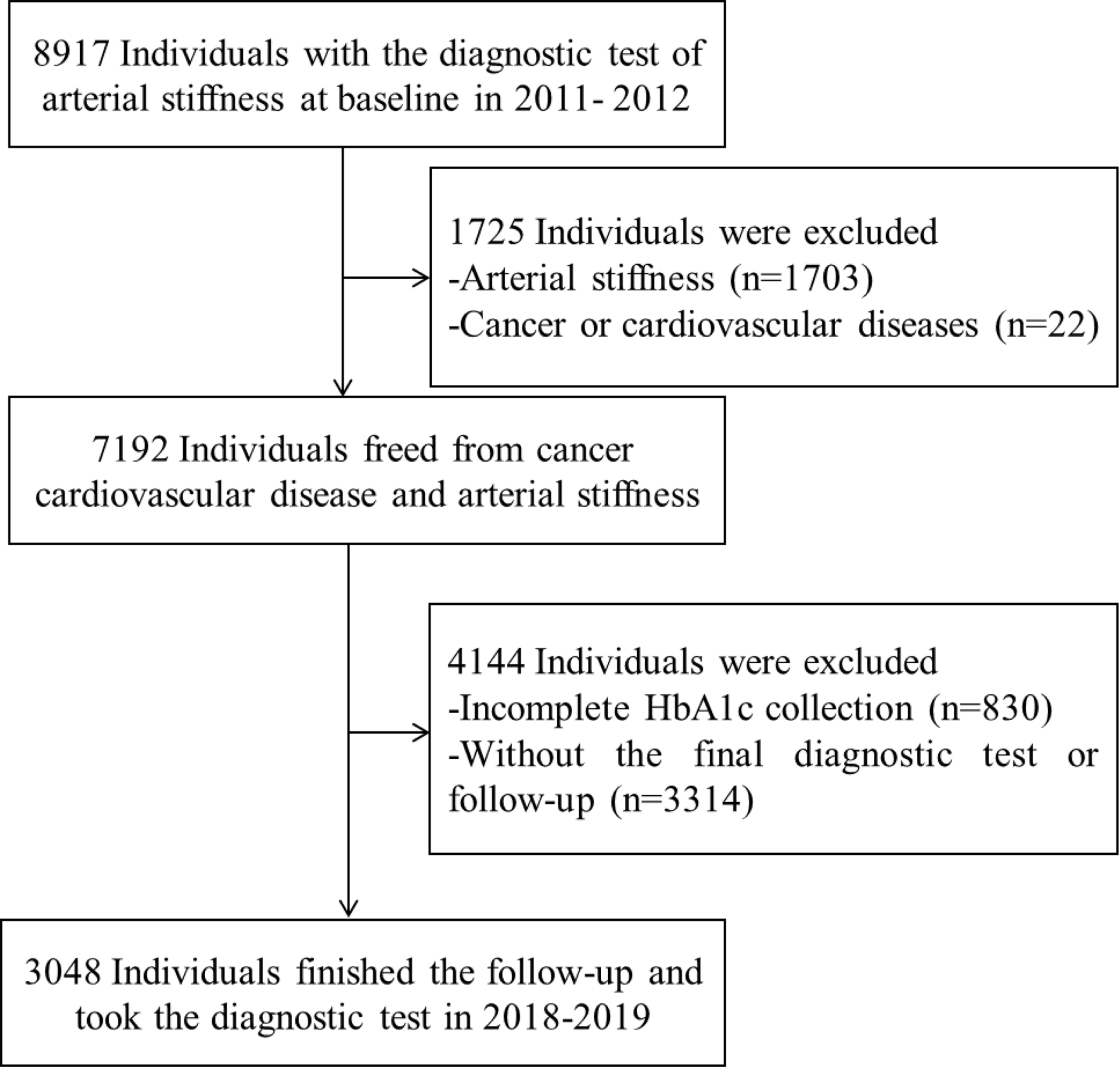
Flow table of the study population in the Beijing Health Management Cohort from 2011–2012 to 2018–2019. HbA1c, glycated hemoglobin.

The Beijing Health Management Cohort (BHMC) study aims to explore the important chronic diseases among participants in Beijing. Participants were enrolled in 2011 and 2012, and followed until December 31, 2019, in this study. Of 8,917 participants, 2,555 participants with cardiovascular diseases, cancer, or atherosclerosis or without information about HbA1c were excluded. A total of 3,314 participants failed to take the final diagnostic test at the final survey. A total of 3,048 participants received at least one follow-up and were enrolled in the study (**Figure 1**).

### Data Collection

Physical and laboratory examination was conducted by trained medical professionals in the whole process based on the 1964 Declaration of Helsinki and the updated version.

Participants were required to take off their shoes and heavy clothes when taking their anthropometric measurements. Body mass index (BMI) is the measure of dividing the weight (kg) by the square of the height (m^2^). After at least 5 min rest and 30 min interval with caffeine forbidden, mean arterial pressure (MAP) was calculated as diastolic blood pressure (DBP) plus one-third of pulse pressure, and the latter was measured as systolic blood pressure (SBP) minus DBP. Participants were supposed to keep calm in the sitting position, and the equipment should be placed at the height of the heart, as well as the tested right arm. Such test should be conducted at least three times with 1–2 min interval, and the mean of the records was applied. After at least 12 h overnight fasting, the blood samples were collected and the following main parameters were tested by high-performance liquid chromatography (HPLC) (chemistry analyzer: Beckman LX 20, Beckman, Brea, CA, USA): HbA1c, FBG, high-density lipoprotein cholesterol (HDL-C), hemoglobin, low-density lipoprotein cholesterol (LDL-C), and triglycerides (TG).

Demographic data were obtained according to the questionnaire. Briefly, information about age, gender, behavioral factors, including physical activity intensity (low, moderate, and high intensity), education level (lower than high-school education and over or equal to high-school education), excessive salt intake (>6 g/day), drinking status (past/current alcohol drinker), smoking status (past/current smoker), sleep duration (<6 h/day, 6–8 h/day and >8 h/day), and medication history of hypertension, diabetes, and hyperlipidemia was collected.

### Definition of Arterial Stiffness

We defined arterial stiffness as baPWV >1,800 m/s or ABI <0.9 (22–24) in this study. The above two indicators were tested with an automatic arterial stiffness analyzer. The requirement of the participants was similar with that when testing the blood pressure. We calculated ABI as the ratio of SBP at the ankle to that on the upper arm on each side, and the minimum ratio was applied. BaPWV was calculated based on pressure in ankles and upper arms and the distance tailored to the height of each participant by the analyzer automatically, and the maximum record was applied.

### Statistical Analysis

We stratified the study population into quartiles based on HbA1c level (Q1 group: participants with HbA1c ≤5.29%; Q2 group: participants with HbA1c in range of 5.30%–5.52%; Q3 group: participants with HbA1c in range of 5.53%–5.81%; Q4 group: participants with HbA1c >5.81%). TyG index was calculated as ln (fasting TG (mg/dl) × FBG (mg/dl)/2). We also classified the participants into quartile groups based on the level of PBG, FBG, and TyG index. Continuous variables were summarized as mean ± standard deviation or median with interquartile range (IQR). Categorical variables were presented as numbers and proportions. We used ANOVA test for non-paired samples of normally distributed parameters and the Kruskal–Wallis test for non-parametric variables. The chi-squared test was applied for the comparison of categorical variables among 'four quartile groups of HbA1c.

Three-step stepwise multivariable-adjusted Cox proportional hazard regression models were conducted to explore the association between HbA1c, PBG, FBG, and TyG index and the risk of the arterial stiffness. Model 1 was adjusted for age and gender. Model 2 was adjusted for variables in model 1, as well as education level, smoking status, drinking status, physical activity intensity, sleep duration, excessive salt intake, anemia, and medication history. Model 3 was adjusted for variables in model 2 plus BMI, MAP, LDL-C, HDL-C, and TG. As for TyG index, we excluded TG from model 3. A multivariable adjusted restricted cubic spline model with 3 knots was used to assess the dose–response relationship between HbA1c, PBG, FBG, and TyG index and the risk of arterial stiffness. Receiver operating characteristic (ROC) analysis was performed to explore the predictive ability of HbA1c, PBG, FBG, and TyG index on arterial stiffness. Considering the effect of glycemic status, ROC curves were also obtained in both diabetic and non-diabetic populations.

We performed several sensitivity analyses. A generalized estimation equation (GEE) model was built to explore the association of repeated measurement of glycemic parameters and arterial stiffness. Cox regression analysis was further performed among diabetics and non-diabetics. Considering the effect of anemia on measurement of HbA1c, Cox regression analysis was conducted and the GEE model was used among individuals without anemia at baseline.

For all analyses, a two-tailed *p*-value <0.05 was considered to be statistically significant. All statistical analyses were performed using R version 3.5.1 (R Foundation for Statistical Computing, Vienna, Austria), SAS version 9.4 (SAS Institute, Cary, North Carolina, USA) and Stata version 15 (College Station, TX, StataCorp LLC).

## Results

### Baseline Characteristics of the Study Population

**Figure 2:**
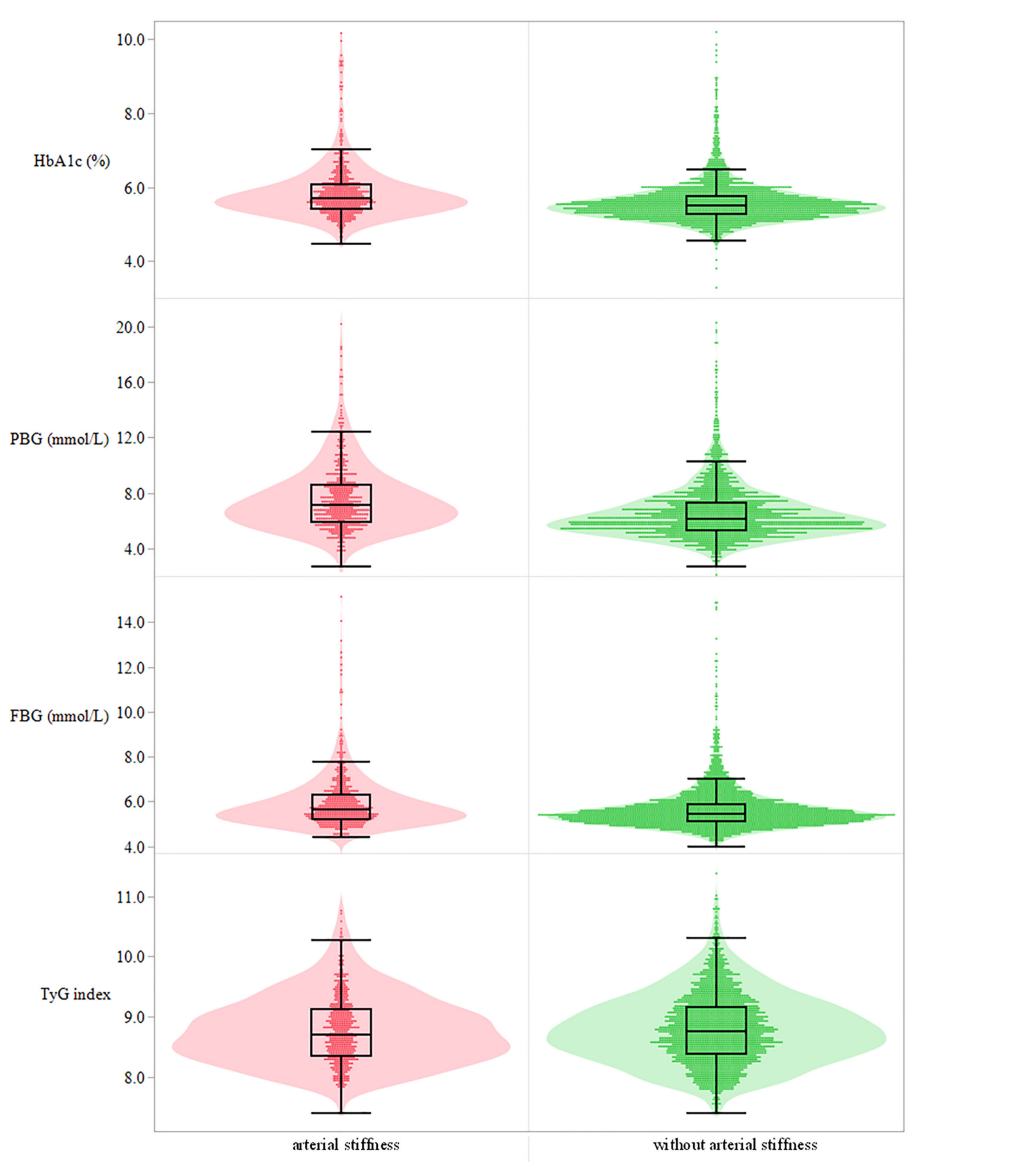
Distribution of the four glucose parameters at baseline among individuals with and without arterial stiffness. Sequentially presented in the picture from top to bottom is HbA1c (%), PBG (mmol/L), FBG (mmol/L), and TyG index. HbA1c, glycated hemoglobin; PBG, postprandial blood glucose; FBG, fasting blood glucose; TyG index, triglyceride-glucose index.

**Figure 3:**
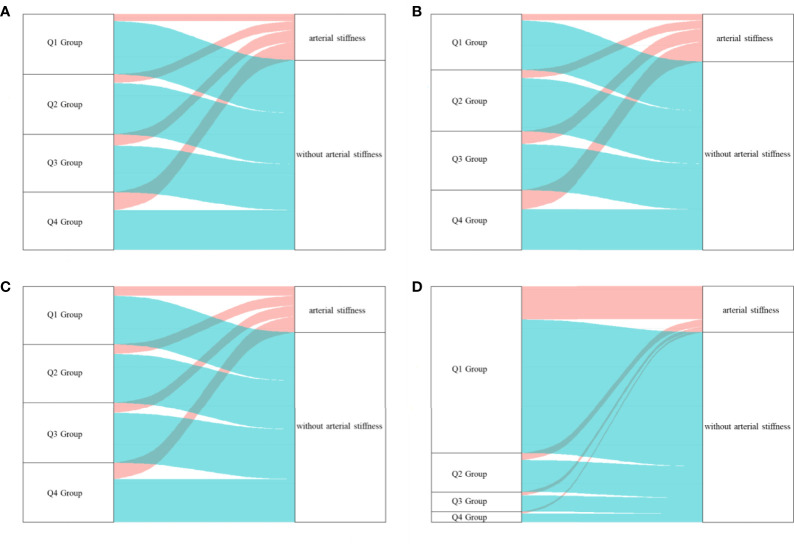
The outcome of participants in different quartile groups of the four glucose parameters at baseline. **(A)** HbA1c, **(B)** PBG, **(C)** FBG, **(D)** TyG index. A large proportion of individuals in higher quartile groups of HbA1c obtained a higher risk of arterial stiffness. HbA1c, glycated hemoglobin; PBG, postprandial blood glucose; FBG, fasting blood glucose; TyG index, triglyceride-glucose index; Q, quartile.

In this cohort study, 3,048 qualified participants finished the follow-up and were enrolled in the final analysis. Of the 3,048 participants, 1,939 participants took the baseline and final examination, and their repeated measurements of glycemic parameters and outcomes in 2013–2017 were also collected. Finally, 1,939 individuals were included to explore the impact of repeated measurement of the glycemic parameters on arterial stiffness. The baseline characteristics of the whole study population in different groups stratified by HbA1c quartiles were summarized in **Table 1**. In detail, 2,311 men and 737 women were investigated. Of the 3,048 participants, 14.93% of the participants were affected by diabetes mellitus and 141 (5.82%) participants were affected by anemia at baseline. The distribution of HbA1c, PBG, FBG, and TyG index at baseline among individuals grouped by the occurrence of arterial stiffness during the follow-up or at the end point is shown in **Figure 2**. The outcome of each participant in different quartile groups of HbA1c, PBG, FBG, and TyG index at baseline is shown in **Figure 3**. The distribution of age, education level, diabetes status, and medication history of hypertension, diabetes, and hyperlipidemia among the quartile groups of HbA1c was significantly different. Individuals in the higher HbA1c quartile group had a significantly higher level of LDL-C, TG, MAP, and BMI and a lower level of HDL-C. A significant difference was not observed among different HbA1c quartile groups for physical activity intensity, smoking status, drinking status, excessive salt intake, anemia, and sleep duration.

**Table 1:** Baseline characteristics of the total participants.

Variables	Total participants	Quartiles of HbA1c				*p-*value
		Q1 (≤5.29%)	Q2 (5.30%–5.52%)	Q3 (5.53%–5.81%)	Q4 (>5.81%)	
No. of participants, *n*	3,048	774	772	748	754	
Arterial stiffness, *n* (%)	591 (19.39)	90 (11.63)	113 (14.64)	149 (19.92)	239 (31.70)	<0.0001
Male, *n* (%)	2,311 (75.82)	582 (75.19)	568 (73.58)	547 (73.13)	614 (81.43)	0.0004
Age (years), median (IQR)	56 (48-63)	45 (52-58)	47 (54-61)	51 (57-64)	54 (59-67)	<0.0001
Age ≥ 65, *n* (%)	667 (21.88)	103 (13.31)	137 (17.75)	182 (24.33)	245 (32.49)	<0.0001
Over or equal to high-school, *n* (%)	2,995 (98.26)	767 (99.10)	762 (98.70)	734 (98.13)	732 (97.08)	0.0165
Physical activity intensity, *n* (%)						0.2358
Low	623 (20.47)	143 (18.48)	152 (19.69)	169 (22.65)	159 (21.14)	
Moderate	2,358 (77.46)	617 (79.72)	599 (77.59)	560 (75.07)	582 (77.39)	
High	63 (2.07)	14 (1.81)	21 (2.72)	17 (2.28)	11 (1.46)	
Past/current smoker, *n* (%)	276 (9.06)	67 (8.66)	61 (7.90)	78 (10.43)	70 (9.28)	0.3681
Past/current alcohol drinker, *n* (%)	374 (12.27)	97 (12.53)	90 (11.66)	97 (12.97)	90 (11.94)	0.8651
Excessive salt intake (>6 g/day), *n* (%)	174 (5.71)	45 (5.81)	30 (3.89)	53 (7.09)	46 (6.10)	0.0543
Sleep duration, *n* (%)						0.3240
<6 h/day	72 (2.36)	16 (2.07)	21 (2.72)	22 (2.94)	13 (1.72)	
6–8 h/day	2,868 (94.36)	731 (94.44)	729 (94.43)	702 (93.85)	706 (93.63)	
>8 h/day	108 (3.54)	27 (3.49)	22 (2.85)	24 (3.21)	35 (4.64)	
Diabetes, *n* (%)	455 (14.93)	11 (1.42)	22 (2.85)	53 (7.09)	369 (48.94)	<0.0001
Anemia, *n* (%)	141 (5.82)	24 (3.98)	34 (5.50)	36 (6.15)	47 (7.62)	0.0550
Medication history of diabetes, *n* (%)	266 (8.73)	6 (0.78)	12 (1.55)	33 (4.41)	215 (28.51)	<0.0001
Medication history of hyperlipidemia, *n* (%)	218 (7.15)	25 (3.23)	38 (4.92)	55 (7.35)	100 (13.26)	<0.0001
Medication history of hypertension, *n* (%)	924 (30.31)	183 (23.64)	196 (25.39)	223 (29.81)	322 (42.71)	<0.0001
MAP (mmHg), median (IQR)	92.67 (84.67–100.33)	92.33 (84.00–100.00)	91.33 (83.67–99.33)	92.00 (84.33–100.00)	94.33 (86.67–102.00)	<0.0001
BMI (kg/m^2^), median (IQR)	25.51 (23.62–27.54)	24.91 (23.26–26.82)	25.25 (23.26–27.19)	25.47 (23.48–27.56)	26.40 (24.55–28.50)	<0.0001
LDL-C (mmol/L) median (IQR),	3.08 (2.52–3.70)	3.01 (2.51–3.59)	3.14 (2.57–3.74)	3.20 (2.64–3.82)	2.98 (2.38–3.65)	<0.0001
TG (mmol/L) median (IQR)	1.39 (1.01–2.05)	1.29 (0.95–1.89)	1.38 (0.98–2.00)	1.42 (1.03–2.06)	1.56 (1.11–2.22)	<0.0001
HDL-C (mmol/L), median (IQR)	1.20 (1.05–1.40)	1.23 (1.07–1.46)	1.22 (1.08–1.41)	1.22 (1.06–1.39)	1.12 (1.04–1.30)	<0.0001

HbA1c, glycated hemoglobin; IQR, interquartile range; Q: quartile; MAP, mean arterial pressure; BMI, body mass index; LDL-C, low-density lipoprotein cholesterol; TG, triglycerides; HDL-C, high-density lipoprotein cholesterol.

### Association Between Arterial Stiffness and Glucose Parameters

**Figure 4:**
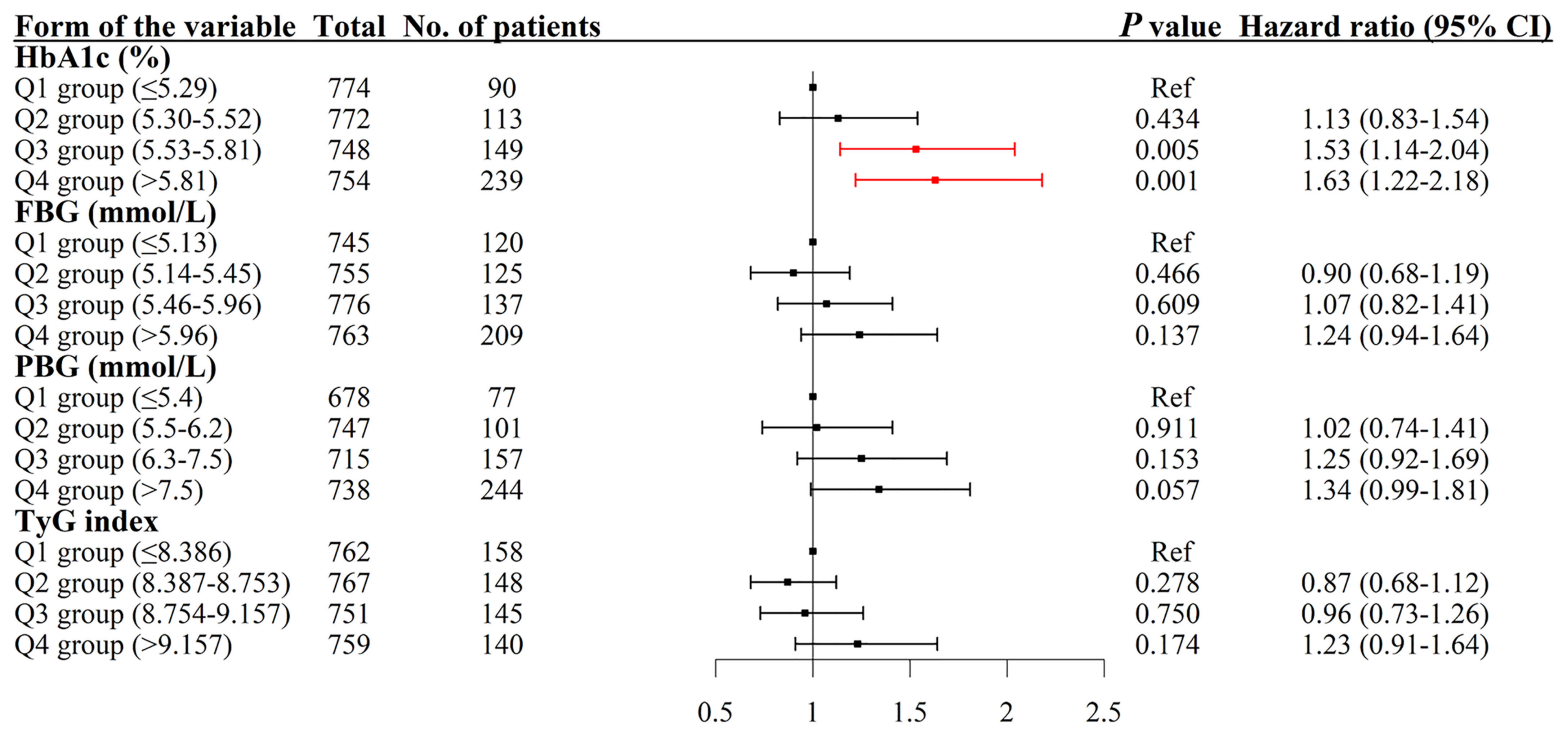
Association between HbA1c, PBG, FBG, and TyG index and the risk of arterial stiffness. The results were robust when more covariates were entered into the model. HbA1c, glycated hemoglobin; Q, quartile; PBG, postprandial blood glucose; FBG, fasting blood glucose; TyG index, triglyceride-glucose index; CI, confidence interval.

The results indicated that compared with the lowest quartile group of HbA1c, people in higher quartile groups had higher HR (**Figure 4**). The HRs (95% CI, *p*-value) were 1.13 (0.83–1.54, *p* = 0.434) for the Q2 group, 1.53 (1.14–2.04, *p* = 0.005) for the Q3 group and 1.63 (1.22–2.18, *p* = 0.001) for the Q4 group with the covariates in Model 3 controlled (**Table 2**).

**Table 2:** Association between glycemic parameters and arterial stiffness among the total population.

Variables	Model 1: HR (95% CI: Lower–Upper)	*p-*value	Model 2: HR (95% CI: Lower–Upper)	*p*-value	Model 3: HR (95% CI: Lower–Upper)	*p-*value	
HbA1c (%)							
Q1 (≤5.29)	Reference		Reference		Reference		
Q2 (5.30–5.52)	1.04 (0.79–1.37)	0.777	1.11 (0.81–1.51)	0.526	1.13 (0.83–1.54)		0.434
Q3 (5.53–5.81)	1.31 (1.01–1.70)	0.045	1.48 (1.11–1.98)	0.008	1.53 (1.14–2.04)		0.005
Q4 (>5.81)	1.69 (1.32–2.16)	<0.001	1.67 (1.25–2.22)	0.001	1.63 (1.22–2.18)		0.001
FBG (mmol/L)							
Q1 (≤5.13)	Reference		Reference		Reference		
Q2 (5.14–5.45)	1.01 (0.79–1.30)	0.917	0.95 (0.72–1.26)	0.733	0.90 (0.68–1.19)		0.466
Q3 (5.46–5.96)	1.09 (0.86–1.40)	0.476	1.14 (0.88–1.49)	0.319	1.07 (0.82–1.41)		0.609
Q4 (>5.96)	1.56 (1.25–1.96)	<0.001	1.45 (1.11–1.90)	0.006	1.24 (0.94–1.64)		0.137
PBG (mmol/L)							
Q1 (≤5.4)	Reference		Reference		Reference		
Q2 (5.5–6.2)	1.06 (0.78–1.42)	0.722	1.10 (0.80–1.51)	0.555	1.02 (0.74–1.41)		0.911
Q3 (6.3–7.5)	1.36 (1.03–1.79)	0.031	1.34 (0.99–1.81)	0.056	1.25 (0.92–1.69)		0.153
Q4 (>7.5)	1.68 (1.29–2.18)	<0.001	1.49 (1.11–2.00)	0.007	1.34 (0.99–1.81)		0.057
TyG index							
Q1 (≤8.386)	Reference		Reference		Reference		
Q2 (8.387–8.753)	1.05 (0.84–1.31)	0.679	0.97 (0.76–1.24)	0.819	0.87 (0.68–1.12)		0.278
Q3 (8.754–9.157)	1.20 (0.95–1.50)	0.122	1.07 (0.83–1.37)	0.592	0.96 (0.73–1.26)		0.750
Q4 (>9.157)	1.64 (1.29–2.08)	<0.001	1.48 (1.14–1.92)	0.003	1.23 (0.91–1.64)		0.174

Model 1: adjusted for age and gender.

Model 2: adjusted for variables in model 1, as well as education level, smoking status, drinking status, physical activity intensity, sleep duration, anemia, excessive salt intake, and medication history of hypertension, diabetes, and hyperlipidemia.

Model 3: adjusted for variables in model 2 plus BMI, MAP, LDL-C, HDL-C, and TG.

HbA1c, glycated hemoglobin; Q, quartile; PBG, postprandial blood glucose; FBG, fasting blood glucose; TyG index, triglyceride-glucose index; HR, hazard ratio; CI, confidence interval.

### Dose–Response Association Between Four Glucose Parameters and Arterial Stiffness

**Figure 5:**
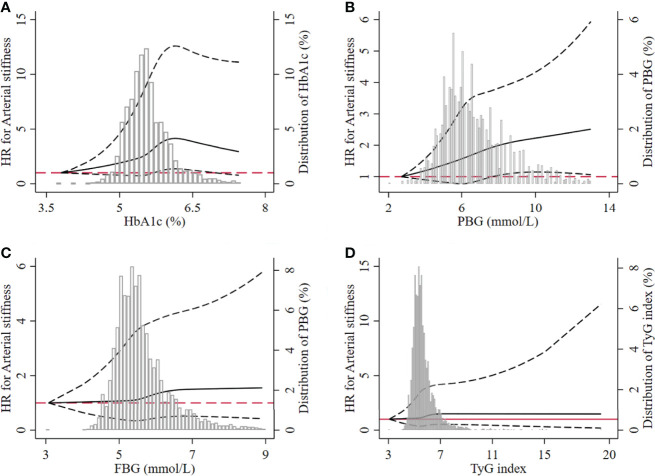
Dose–response relationship between the four glucose parameters and the risk of arterial stiffness. **(A)** HbA1c, **(B)** PBG, **(C)** FBG, **(D)** TyG index. The dotted lines represented the lower and upper limit of 95% CI at each dot. HbA1c, glycated hemoglobin; PBG, postprandial blood glucose; FBG, fasting blood glucose; TyG index, triglyceride-glucose index; HR, hazard ratio.

The result of the restricted cubic spline model showed that the level of HbA1c from 5.71% to 6.95% had a significantly nonlinear relationship with the risk of arterial stiffness. The nonlinear relationship between arterial stiffness and PBG level higher than or equal to 7.70 mmol/L was observed. However, we did not find such relationship between FBG and TyG index and the risk of arterial stiffness (**Figure 5**).

### Predictive Ability of Four Glucose Parameters for Arterial Stiffness

**Figure 6:**
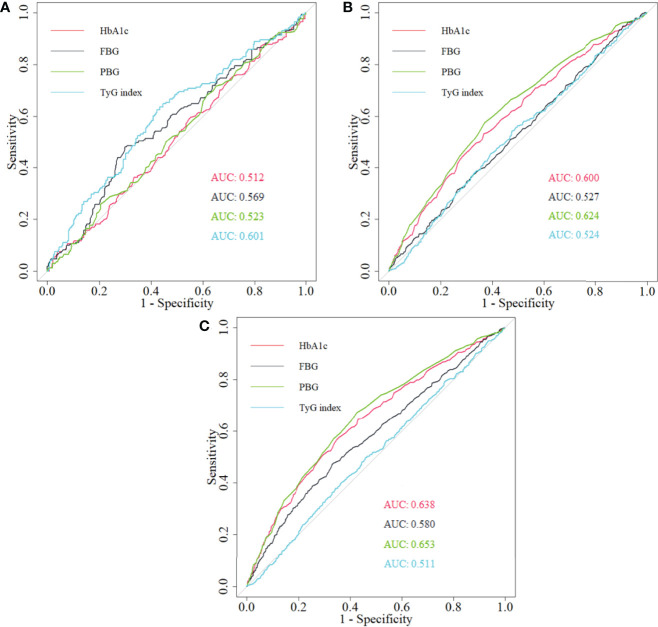
The comparison of the predictive ability of HbA1c, PBG, FBG, and TyG index for arterial stiffness among **(A)** diabetics, **(B)** non-diabetics, and **(C)** total population. HbA1c, glycated hemoglobin; PBG, postprandial blood glucose; FBG, fasting blood glucose; TyG index, triglyceride-glucose index; ROC, receiver operating characteristic; AUC, area under curve.

HbA1c had powerful predictive ability on arterial stiffness among non-diabetics and the whole study population (**Figure 6**). Although PBG presented a better predictive performance, statistical significance was not observed when comparing areas under curve (AUCs) between PBG and HbA1c (**Supplementary Table S1**).

### Sensitivity Analysis

HbA1c was the strongest risk factor for arterial stiffness (HR: 1.47, 95% CI: 1.25–1.72) in comparison to other parameters (**Supplementary Table S2**) based on GEE analysis. The significant association was found between HbA1c and the risk of arterial stiffness among non-diabetics (**Supplementary Table S3**), but such association was not found among diabetics (**Supplementary Table S4**). When individuals with an anemic status at baseline were excluded, the robust results were obtained. Individuals in the higher quartile group of HbA1c had a higher risk for arterial stiffness (**Supplementary Table S5**), and the association between repeated measurement of HbA1c and the higher risk of arterial stiffness was statistically significant (**Supplementary Table S6**). Such significant association was observed among the non-diabetic population without anemia at baseline (**Supplementary Table S7**), but not in the diabetic population without anemia at baseline (**Supplementary Table S8**).

## Discussion

In our longitudinal study, HbA1c had a significantly positive relationship with the risk of arterial stiffness compared with PBG, FBG, and TyG index. The nonlinear relationship was found between HbA1c in the level from 5.71% to 6.95% and the risk of arterial stiffness. HbA1c and PBG had better predictive performance of arterial stiffness among the whole and non-diabetic population. The robust results were obtained.

Our result illustrated the important effect of HbA1c on arterial stiffness partly because of the glycation process (25). Hyperglycemia and insulin resistance could accelerate the progression of arterial lesions (26, 27). Insulin resistance can be regarded as treating target of arterial stiffness with the significant reduction of HbA1c among diabetics (28). Moreno (29) found that HbA1c could independently express the differences in the risk of arterial stiffness between groups with controlled and uncontrolled diabetes mellitus. Johansen (28) demonstrated that changes in HbA1c over time or measurement at baseline both had an impact on aortic stiffness. HbA1c is an indicator of long-term variation of blood glucose and advanced glycation products. Chronic hyperglycemia with long-term high level of HbA1c can promote protein glycosylation and accelerate the progression of arterial stiffness (29). In the long process of glycation, intermediate and end glycation products would result in the pathological change of arterial wall and endothelial dysfunction, and accelerate the progression of arterial stiffness (30).

In our study, we found that HbA1c is an effective risk factor of arterial stiffness compared with three other glucose parameters. Some previous studies had investigated the effect of TyG index, an essential indicator of insulin-resistance, PBG, and FBG on arterial stiffness in different groups of population (31–33). However, comparison between these parameters was seldom conducted, and HbA1c had not been intensively explored. Our study supplies practical information about comparison of the risk among these glucose parameters on arterial stiffness based on existing mechanisms (34, 35). PBG level higher than or equal to 7.70 mmol/L and the level of HbA1c from 4% to 6% can be treated as abnormal glycemic status. Thus, our results of the nonlinear relationship of PBG ≥7.70 mmol/L and HbA1c from 5.71% to 6.95% with arterial stiffness were reliable.

The predictive role of HbA1c for arterial stiffness was proved previously (36), and the better predictive ability of HbA1c was found compared with FBG (10) in both diabetic and non-diabetic groups. The result was similar with ours. The better predictive ability of TyG index on arterial stiffness among diabetics was obtained, because TyG index was strongly associated with glycemic status and insulin resistance and was a better indicator of diabetic status (37–39). The better predictive role of PBG among non-diabetics and the whole study population was found compared with HbA1c; however, a significant difference was not found. Moreover, PBG was affected by personal diet pattern and not easily accessible. Although the significant association between HbA1c and arterial stiffness was not observed among diabetics, it is acknowledged that HbA1C is a strong prospective tool to assess the risk of diabetic complications and an effective indicator of treatment of diabetes (40, 41). Thus, the predictive role of HbA1c on arterial stiffness should not be ignored.

In many studies, the positive association between HbA1c and arteriosclerosis disappeared (42) after adjusting for general confounding factors. The endpoint of our study is the occurrence of arterial stiffness diagnosed by baPWV and ABI. Many studies had diagnosed arterial stiffness by angiography examination. Angiography examination can express the progression and condition of arteriosclerosis (43), but its measurement was not sensitive at the initial stage of arteriosclerosis. BaPWV and ABI are simple and harmless tests and can serve as a routine clinical examination for measuring arterial stiffness (24). The differences in diagnosis criteria among these studies would cause contradictory results. On the other hand, measurement of HbA1c was different, the sample sizes of the study populations were distinct (11), and the study population was composed of individuals with different characteristics (44). Health condition and medication history of the study population also had an impact. Statin therapy, one of the most widely applied dyslipidemia drugs (45), would improve endothelial function (46) and further improve the condition of vascular sclerosis. Diabetes drugs would preserve anti-oxidant function, and further decrease platelet activation and aggregation (47). In our analysis, medication history was taken into consideration as a confounding factor. Thus, the confounding effects of medication history were properly adjusted. More detailed information about medication history, such as the specific type, will be further investigated.

There are several strengths of this study. It should be emphasized that this is a longitudinal study investigating the positive association of baseline and repeated measurement of HbA1c and arterial stiffness. Besides HbA1c, we also compared the impact of PBG, FBG, and TyG index on arterial stiffness. ROC analysis was performed, and the results illustrated the highest risk of abnormal level of HbA1c and its strong predictive ability on arterial stiffness. Multiple sensitivity analyses are also the strength, and the robust results were obtained.

The present study has some limitations. First, we included all qualified individuals to make our conclusion accessible to the general population. However, more participants from different nationalities or races should be enrolled. Second, although HPLC is an effective method to differentiate HbA1c and some types of Hb variants, the influence of Hb variants on measurement of HbA1c should not be ignored. Many glycemic parameters, such as glycemic variability and time in range, have the potential to reflect the dynamic change in blood glucose (48, 49). Such parameters would better reflect glycemic status. Thus, we can investigate the impact of these parameters on the progression of arterial stiffness in future studies.

## Conclusions

In this longitudinal study, the association between HbA1c and the higher risk of arterial stiffness among the general or non-diabetic population was observed compared with PBG, FBG, and TyG index assessed by baPWV and ABI among a Chinese Han population. This study suggests the necessity for the early detection and management of arterial stiffness among a population with a high abnormal level of HbA1c, especially among non-diabetics.

## Data Availability Statement

The raw data supporting the conclusions of this article will be made available by the authors, without undue reservation.

## Ethics Statement

The studies involving human participants were reviewed and approved by the Ethics Committee of the Capital Medical University (number 2013SY26). The patients/participants provided written informed consent to participate in this study.

## Author Contributions

ZH, LT, and SC came up with the idea and made the design of the study. XK contributed to the data. ZH and JW conducted the statistical analysis. ZH drafted the primary manuscript. ZH, JL, JZ, JW, XZ, YL, and ZW revised the manuscript and provided important advice for modification. All authors contributed to the article and approved the submitted version.

## Funding

This work was supported by the National Natural Science Foundation of China (grant numbers 82073668 and 81872708).

## Conflict of Interest

The authors declare that the research was conducted in the absence of any commercial or financial relationships that could be construed as a potential conflict of interest.

## Publisher’s Note

All claims expressed in this article are solely those of the authors and do not necessarily represent those of their affiliated organizations, or those of the publisher, the editors and the reviewers. Any product that may be evaluated in this article, or claim that may be made by its manufacturer, is not guaranteed or endorsed by the publisher.

## Abbreviations

HbA1c, glycated hemoglobin; FBG, fasting blood glucose; PBG, postprandial blood glucose; TyG Index, triglyceride-glucose index; BHMC, Beijing Health Management Cohort; baPWV, brachial-ankle pulse wave velocity; ABI, ankle brachial index; BMI, body mass index; MAP, mean arterial pressure; DBP, diastolic blood pressure; SBP, systolic blood pressure; HDL-C, high-density lipoprotein cholesterol; LDL-C, low-density lipoprotein cholesterol; TG, triglycerides; HR, hazard ratio; IQR, interquartile range; CI, confidence interval; Q, quartile; HPLC, high-performance liquid chromatography; ROC, receiver operating characteristic; AUC, area under curve.
