# Supplementary material for: Glycated Hemoglobin and Risk of Arterial Stiffness in a Chinese Han Population: A Longitudinal Study
Source: Front Endocrinol (Lausanne). 2022 Apr 29;13:854875. doi: 10.3389/fendo.2022.854875 (PMC9098962; doi:10.3389/fendo.2022.854875)
Supplement: Supplementary file 1 [file Table_1.docx]

| Table S1. Predictive ability of glycemic parameters for arterial stiffness | | | | | | | |  |
| --- | --- | --- | --- | --- | --- | --- | --- | --- |
|  |  |  |  | Comparison of AUCs between the glycemic parameters (*P* value) | | | |  |
| Variables | AUC | 95% CI | *P* value | HbA1c | FBG | PBG | TyG index | |
| Total population |  |  |  |  |  |  |  | |
| HbA1c (%) | 0.638 | (0.613-0.664) | <0.001 | - | <0.001 | 0.412 | <0.001 | |
| FBG (mmol/L) | 0.580 | (0.554-0.607) | <0.001 |  | - | <0.001 | <0.001 | |
| PBG (mmol/L) | 0.653 | (0.628-0.678) | <0.001 |  |  | - | <0.001 | |
| TyG index | 0.511 | (0.485-0.537) | 0.400 |  |  |  | - | |
| Non-diabetic population |  |  |  |  |  |  |  | |
| HbA1c (%) | 0.600 | (0.570-0.630) | <0.001 | - | <0.001 | 0.275 | <0.001 | |
| FBG (mmol/L) | 0.527 | (0.497-0.557) | 0.075 |  | - | <0.001 | 0.867 | |
| PBG (mmol/L) | 0.624 | (0.594-0.653) | <0.001 |  |  | - | <0.001 | |
| TyG index | 0.524 | (0.494-0.554) | 0.120 |  |  |  | - | |
| Diabetic population |  |  |  |  |  |  |  | |
| HbA1c (%) | 0.512 | (0.457-0.567) | 0.677 | - | 0.251 | 0.782 | 0.049 | |
| FBG (mmol/L) | 0.569 | (0.514-0.624) | 0.014 |  | - | 0.247 | 0.222 | |
| PBG (mmol/L) | 0.523 | (0.467-0.579) | 0.425 |  |  | - | 0.043 | |
| TyG index | 0.601 | (0.547-0.656) | <0.001 |  |  |  | - | |
| HbA1c, glycated hemoglobin; Q, quartile; PBG, postprandial blood glucose; FBG, fasting blood glucose; TyG index, triglyceride-glucose index; HR, hazard ratio; CI, confidence interval; AUC, area under curve. | | | | | | | | |

| Table S2. Association between repeated measurement of glycemic parameters and arterial stiffness among total population | | | | | | |
| --- | --- | --- | --- | --- | --- | --- |
| Variables | Model 1: HR (95% CI: Lower-Upper) | *P* value | Model 2: HR (95% CI: Lower-Upper) | *P* value | Model 3: HR (95% CI: Lower-Upper) | *P* value |
| HbA1c (%) | 1.48  (1.33-1.65) | <0.001 | 1.47  (1.28-1.69) | <0.001 | 1.47  (1.25-1.72) | <0.001 |
| FBG (mmol/L) | 1.14  (1.05-1.24) | 0.001 | 1.14  (1.03-1.26) | 0.012 | 1.09  (0.98-1.23) | 0.117 |
| PBG (mmol/L) | 1.10  (1.06-1.15) | <0.001 | 1.08  (1.03-1.12) | 0.001 | 1.09  (1.04-1.14) | 0.001 |
| TyG index | 1.52  (1.30-1.78) | <0.001 | 1.49  (1.25-1.78) | <0.001 | 1.41  (1.14-1.74) | 0.002 |
| The glycemic parameters were modelled as continuous variables.  Model 1: adjusted for age and gender.  Model 2: adjusted for variables in model 1, as well as education level, smoking status, drinking status, physical activity intensity, sleep duration, anemia, excessive salt intake and medication history of hypertension, diabetes and hyperlipidemia.  Model 3: adjusted for variables in model 2 plus BMI, MAP, LDL-C, HDL-C and TG.  HbA1c, glycated hemoglobin; Q, quartile; PBG, postprandial blood glucose; FBG, fasting blood glucose; TyG index, triglyceride-glucose index; HR, hazard ratio; CI, confidence interval. | | | | | | |

| Table S3. Association between glycemic parameters and arterial stiffness among non-diabetics | | | | | | | |
| --- | --- | --- | --- | --- | --- | --- | --- |
| Variables | Model 1: HR (95% CI: Lower-Upper) | *P* value | Model 2: HR (95% CI: Lower-Upper) | *P* value | Model 3: HR (95% CI: Lower-Upper) | *P* value | |
| HbA1c (%) |  | |  | |  | | |
| Q1 (≤5.29) | Reference | | Reference | | Reference | | |
| Q2 (5.30-5.52) | 1.05 (0.78-1.39) | 0.766 | 1.13 (0.82-1.56) | 0.465 | 1.15 (0.83-1.58) | | 0.406 |
| Q3 (5.53-5.81) | 1.29 (0.98-1.70) | 0.070 | 1.55 (1.14-2.11) | 0.005 | 1.59 (1.17-2.17) | | 0.003 |
| Q4 (>5.81) | 1.40 (1.04-1.89) | 0.025 | 1.66 (1.20-2.29) | 0.002 | 1.67 (1.20-2.31) | | 0.002 |
| FBG (mmol/L) |  | |  | |  | | |
| Q1 (≤5.13) | Reference | | Reference | | Reference | | |
| Q2 (5.14-5.45) | 1.04 (0.80-1.35) | 0.753 | 0.97 (0.73-1.29) | 0.827 | 0.93 (0.69-1.24) | | 0.603 |
| Q3 (5.46-5.96) | 1.09 (0.84-1.41) | 0.518 | 1.12 (0.84-1.48) | 0.437 | 1.13 (0.85-1.51) | | 0.392 |
| Q4 (>5.96) | 1.27 (0.94-1.72) | 0.125 | 1.30 (0.93-1.80) | 0.122 | 1.19 (0.84-1.68) | | 0.323 |
| PBG (mmol/L) |  | |  | |  | | |
| Q1 (≤5.4) | Reference | | Reference | | Reference | | |
| Q2 (5.5-6.2) | 1.14 (0.83-1.55) | 0.425 | 1.17 (0.84-1.65) | 0.354 | 1.13 (0.80-1.59) | | 0.500 |
| Q3 (6.3-7.5) | 1.39 (1.03-1.86) | 0.030 | 1.36 (0.99-1.89) | 0.062 | 1.34 (0.96-1.86) | | 0.082 |
| Q4 (>7.5) | 1.37 (1.00-1.86) | 0.050 | 1.39 (0.99-1.95) | 0.060 | 1.33 (0.94-1.88) | | 0.112 |
| TyG index |  | |  | |  | | |
| Q1 (≤8.386) | Reference | | Reference | | Reference | | |
| Q2 (8.387-8.753) | 1.05 (0.82-1.35) | 0.713 | 0.99 (0.75-1.30) | 0.922 | 0.90 (0.68-1.19) | | 0.454 |
| Q3 (8.754-9.157) | 1.09 (0.83-1.44) | 0.515 | 1.12 (0.84-1.51) | 0.437 | 1.03 (0.75-1.41) | | 0.880 |
| Q4 (>9.157) | 1.52 (1.14-2.03) | 0.004 | 1.42 (1.02-1.96) | 0.035 | 1.19 (0.83-1.69) | | 0.340 |
| Model 1: adjusted for age and gender.  Model 2: adjusted for variables in model 1, as well as education level, smoking status, drinking status, physical activity intensity, sleep duration, anemia, excessive salt intake and medication history of hypertension, diabetes and hyperlipidemia.  Model 3: adjusted for variables in model 2 plus BMI, MAP, LDL-C, HDL-C and TG.  HbA1c, glycated hemoglobin; Q, quartile; PBG, postprandial blood glucose; FBG, fasting blood glucose; TyG index, triglyceride-glucose index; HR, hazard ratio; CI, confidence interval. | | | | | | | |

| Table S4. Association between glycemic parameters and arterial stiffness among diabetics | | | | | | | |
| --- | --- | --- | --- | --- | --- | --- | --- |
| Variables | Model 1: HR (95% CI: Lower-Upper) | *P* value | Model 2: HR (95% CI: Lower-Upper) | *P* value | Model 3: HR (95% CI: Lower-Upper) | *P* value | |
| HbA1c (%) |  | |  | |  | | |
| Q1 (≤5.29) | Reference | | Reference | | Reference | | |
| Q2 (5.30-5.52) | 0.61 (0.20-1.91) | 0.395 | 0.61 (0.19-1.94) | 0.402 | 0.74 (0.23-2.43) | | 0.619 |
| Q3 (5.53-5.81) | 0.71 (0.28-1.80) | 0.475 | 0.66 (0.26-1.72) | 0.398 | 0.72 (0.27-1.90) | | 0.504 |
| Q4 (>5.81) | 0.76 (0.33-1.72) | 0.503 | 0.68 (0.29-1.59) | 0.377 | 0.72 (0.29-1.77) | | 0.476 |
| FBG (mmol/L) |  | |  | |  | | |
| Q1 (≤5.13) | Reference | | Reference | | Reference | | |
| Q2 (5.14-5.45) | 0.47 (0.18-1.20) | 0.113 | 0.52 (0.18-1.47) | 0.218 | 0.41 (0.14-1.20) | | 0.103 |
| Q3 (5.46-5.96) | 0.69 (0.29-1.66) | 0.410 | 0.67 (0.27-1.70) | 0.403 | 0.58 (0.23-1.47) | | 0.249 |
| Q4 (>5.96) | 0.72 (0.35-1.47) | 0.362 | 0.75 (0.34-1.63) | 0.464 | 0.60 (0.27-1.33) | | 0.212 |
| PBG (mmol/L) |  | |  | |  | | |
| Q1 (≤5.4) | Reference | | Reference | | Reference | | |
| Q2 (5.5-6.2) | 0.39 (0.14-1.07) | 0.068 | 0.41 (0.15-1.15) | 0.089 | 0.43 (0.15-1.20) | | 0.107 |
| Q3 (6.3-7.5) | 0.67 (0.31-1.43) | 0.295 | 0.68 (0.31-1.50) | 0.340 | 0.63 (0.28-1.38) | | 0.244 |
| Q4 (>7.5) | 0.77 (0.40-1.46) | 0.417 | 0.76 (0.40-1.47) | 0.417 | 0.66 (0.34-1.28) | | 0.217 |
| TyG index |  | |  | |  | | |
| Q1 (≤8.386) | Reference | | Reference | | Reference | | |
| Q2 (8.387-8.753) | 0.91 (0.55-1.51) | 0.727 | 0.77 (0.45-1.32) | 0.337 | 0.69 (0.40-1.19) | | 0.179 |
| Q3 (8.754-9.157) | 1.10 (0.70-1.74) | 0.674 | 0.91 (0.55-1.50) | 0.713 | 0.81 (0.47-1.40) | | 0.450 |
| Q4 (>9.157) | 1.12 (0.68-1.83) | 0.659 | 1.11 (0.65-1.89) | 0.704 | 0.97 (0.54-1.75) | | 0.930 |
| Model 1: adjusted for age and gender.  Model 2: adjusted for variables in model 1, as well as education level, smoking status, drinking status, physical activity intensity, sleep duration, anemia, excessive salt intake and medication history of hypertension, diabetes and hyperlipidemia.  Model 3: adjusted for variables in model 2 plus BMI, MAP, LDL-C, HDL-C and TG.  HbA1c, glycated hemoglobin; Q, quartile; PBG, postprandial blood glucose; FBG, fasting blood glucose; TyG index, triglyceride-glucose index; HR, hazard ratio; CI, confidence interval. | | | | | | | |

| Table S5. Association between glycemic parameters and arterial stiffness among participants without anemia at baseline | | | | | | | | |  |
| --- | --- | --- | --- | --- | --- | --- | --- | --- | --- |
| Variables | Model 1: HR (95% CI: Lower-Upper) | *P* value | Model 2: HR (95% CI: Lower-Upper) | *P* value | Model 3: HR (95% CI: Lower-Upper) | *P* value | | | |
| HbA1c (%) |  | |  | |  | | | | |
| Q1 (≤5.29) | Reference | | Reference | | Reference | | | | |
| Q2 (5.30-5.52) | 1.04 (0.78-1.39) | 0.774 | 1.05 (0.79-1.40) | 0.725 | 1.09 (0.82-1.45) | | 0.570 | | |
| Q3 (5.53-5.81) | 1.23 (0.94-1.61) | 0.139 | 1.23 (0.94-1.61) | 0.141 | 1.27 (0.96-1.66) | | 0.092 | | |
| Q4 (>5.81) | 1.67 (1.30-2.16) | <0.001 | 1.46 (1.11-1.90) | 0.006 | 1.42 (1.08-1.86) | | 0.011 | | |
| FBG (mmol/L) |  | |  | |  | | | | |
| Q1 (≤5.13) | Reference | | Reference | | Reference | | | | |
| Q2 (5.14-5.45) | 1.00 (0.77-1.31) | 0.986 | 0.99 (0.76-1.29) | 0.945 | 0.93 (0.71-1.22) | | 0.609 | | |
| Q3 (5.46-5.96) | 1.10 (0.85-1.42) | 0.479 | 1.04 (0.80-1.35) | 0.772 | 0.98 (0.75-1.28) | | 0.882 | | |
| Q4 (>5.96) | 1.56 (1.23-1.98) | <0.001 | 1.29 (0.99-1.67) | 0.058 | 1.10 (0.84-1.44) | | 0.502 | | |
| PBG (mmol/L) |  | |  | |  | | | | |
| Q1 (≤5.4) | Reference | | Reference | | Reference | | | | |
| Q2 (5.5-6.2) | 1.02 (0.75-1.39) | 0.898 | 0.98 (0.72-1.33) | 0.871 | 0.90 (0.66-1.23) | | 0.504 | | |
| Q3 (6.3-7.5) | 1.40 (1.05-1.85) | 0.020 | 1.32 (1.00-1.76) | 0.052 | 1.25 (0.94-1.66) | | 0.133 | | |
| Q4 (>7.5) | 1.73 (1.32-2.27) | <0.001 | 1.43 (1.08-1.89) | 0.012 | 1.30 (0.98-1.73) | | 0.070 | | |
| TyG index |  | |  | |  | | | | |
| Q1 (≤8.386) | Reference | | Reference | | Reference | | | | |
| Q2 (8.387-8.753) | 1.04 (0.82-1.32) | 0.720 | 0.97 (0.76-1.23) | 0.802 | 0.87 (0.68-1.12) | | 0.278 | | |
| Q3 (8.754-9.157) | 1.21 (0.95-1.54) | 0.122 | 1.09 (0.85-1.39) | 0.501 | 0.95 (0.73-1.23) | | 0.687 | | |
| Q4 (>9.157) | 1.60 (1.25-2.06) | <0.001 | 1.42 (1.10-1.83) | 0.007 | 1.14 (0.86-1.52) | | 0.351 | | |
| Model 1: adjusted for age and gender.  Model 2: adjusted for variables in model 1, as well as education level, smoking status, drinking status, physical activity intensity, sleep duration, anemia, excessive salt intake and medication history of hypertension, diabetes and hyperlipidemia.  Model 3: adjusted for variables in model 2 plus BMI, MAP, LDL-C, HDL-C and TG.  HbA1c, glycated hemoglobin; Q, quartile; PBG, postprandial blood glucose; FBG, fasting blood glucose; TyG index, triglyceride-glucose index; HR, hazard ratio; CI, confidence interval. | | | | | | | |  |  |

| Table S6. Association between repeated measurement of glycemic parameters and arterial stiffness among participants without anemia at baseline | | | | | | |
| --- | --- | --- | --- | --- | --- | --- |
| Variables | Model 1: HR (95% CI: Lower-Upper) | *P* value | Model 2: HR (95% CI: Lower-Upper) | *P* value | Model 3: HR (95% CI: Lower-Upper) | *P* value |
| HbA1c (%) | 1.48  (1.33-1.65) | <0.001 | 1.40  (1.25-1.58) | <0.001 | 1.39  (1.22-1.59) | <0.001 |
| FBG (mmol/L) | 1.14  (1.05-1.24) | 0.002 | 1.08  (0.99-1.18) | 0.099 | 1.04  (0.94-1.15) | 0.446 |
| PBG (mmol/L) | 1.10  (1.06-1.14) | <0.001 | 1.07  (1.03-1.12) | <0.001 | 1.09  (1.04-1.13) | <0.001 |
| TyG index | 1.50  (1.28-1.76) | <0.001 | 1.41  (1.20-1.66) | <0.001 | 1.26  (1.04-1.53) | 0.018 |
| The glycemic parameters were modelled as continuous variables.  Model 1: adjusted for age and gender.  Model 2: adjusted for variables in model 1, as well as education level, smoking status, drinking status, physical activity intensity, sleep duration, anemia, excessive salt intake and medication history of hypertension, diabetes and hyperlipidemia.  Model 3: adjusted for variables in model 2 plus BMI, MAP, LDL-C, HDL-C and TG.  HbA1c, glycated hemoglobin; Q, quartile; PBG, postprandial blood glucose; FBG, fasting blood glucose; TyG index, triglyceride-glucose index; HR, hazard ratio; CI, confidence interval. | | | | | | |

| Table S7. Association between glycemic parameters and arterial stiffness among non-diabetics without anemia at baseline | | | | | | | |
| --- | --- | --- | --- | --- | --- | --- | --- |
| Variables | Model 1: HR (95% CI: Lower-Upper) | *P* value | Model 2: HR (95% CI: Lower-Upper) | *P* value | Model 3: HR (95% CI: Lower-Upper) | *P* value | |
| HbA1c (%) |  | |  | |  | | |
| Q1 (≤5.29) | Reference | | Reference | | Reference | | |
| Q2 (5.30-5.52) | 1.05 (0.78-1.41) | 0.741 | 1.07 (0.80-1.44) | 0.646 | 1.10 (0.82-1.49) | | 0.533 |
| Q3 (5.53-5.81) | 1.21 (0.91-1.61) | 0.194 | 1.26 (0.95-1.67) | 0.116 | 1.28 (0.96-1.71) | | 0.093 |
| Q4 (>5.81) | 1.41 (1.04-1.91) | 0.028 | 1.42 (1.04-1.92) | 0.025 | 1.42 (1.04-1.95) | | 0.028 |
| FBG (mmol/L) |  | |  | |  | | |
| Q1 (≤5.13) | Reference | | Reference | | Reference | | |
| Q2 (5.14-5.45) | 1.05 (0.79-1.38) | 0.756 | 1.03 (0.78-1.36) | 0.833 | 0.98 (0.74-1.29) | | 0.870 |
| Q3 (5.46-5.96) | 1.11 (0.85-1.45) | 0.450 | 1.07 (0.82-1.40) | 0.633 | 1.04 (0.79-1.37) | | 0.806 |
| Q4 (>5.96) | 1.26 (0.92-1.72) | 0.147 | 1.23 (0.89-1.68) | 0.207 | 1.07 (0.77-1.48) | | 0.694 |
| PBG (mmol/L) |  | |  | |  | | |
| Q1 (≤5.4) | Reference | | Reference | | Reference | | |
| Q2 (5.5-6.2) | 1.07 (0.78-1.47) | 0.694 | 1.01 (0.73-1.39) | 0.965 | 0.95 (0.68-1.32) | | 0.750 |
| Q3 (6.3-7.5) | 1.38 (1.03-1.86) | 0.034 | 1.34 (0.99-1.81) | 0.056 | 1.29 (0.95-1.75) | | 0.104 |
| Q4 (>7.5) | 1.43 (1.05-1.96) | 0.025 | 1.33 (0.97-1.82) | 0.079 | 1.26 (0.91-1.73) | | 0.166 |
| TyG index |  | |  | |  | | |
| Q1 (≤8.386) | Reference | | Reference | | Reference | | |
| Q2 (8.387-8.753) | 1.09 (0.84-1.42) | 0.531 | 1.03 (0.79-1.34) | 0.849 | 0.92 (0.70-1.22) | | 0.571 |
| Q3 (8.754-9.157) | 1.13 (0.85-1.50) | 0.407 | 1.08 (0.81-1.44) | 0.589 | 0.96 (0.71-1.30) | | 0.791 |
| Q4 (>9.157) | 1.55 (1.15-2.08) | 0.004 | 1.47 (1.09-1.98) | 0.013 | 1.20 (0.86-1.66) | | 0.286 |
| Model 1: adjusted for age and gender.  Model 2: adjusted for variables in model 1, as well as education level, smoking status, drinking status, physical activity intensity, sleep duration, anemia, excessive salt intake and medication history of hypertension, diabetes and hyperlipidemia.  Model 3: adjusted for variables in model 2 plus BMI, MAP, LDL-C, HDL-C and TG.  HbA1c, glycated hemoglobin; Q, quartile; PBG, postprandial blood glucose; FBG, fasting blood glucose; TyG index, triglyceride-glucose index; HR, hazard ratio; CI, confidence interval. | | | | | | | |

| Table S8. Association between glycemic parameters and arterial stiffness among diabetics without anemia at baseline | | | | | | | |
| --- | --- | --- | --- | --- | --- | --- | --- |
| Variables | Model 1: HR (95% CI: Lower-Upper) | *P* value | Model 2: HR (95% CI: Lower-Upper) | *P* value | Model 3: HR (95% CI: Lower-Upper) | *P* value | |
| HbA1c (%) |  | |  | |  | | |
| Q1 (≤5.29) | Reference | | Reference | | Reference | | |
| Q2 (5.30-5.52) | 0.61 (0.20-1.92) | 0.402 | 0.61 (0.19-1.96) | 0.411 | 0.72 (0.22-2.35) | | 0.583 |
| Q3 (5.53-5.81) | 0.71 (0.28-1.82) | 0.476 | 0.64 (0.24-1.68) | 0.364 | 0.68 (0.25-1.84) | | 0.452 |
| Q4 (>5.81) | 0.78 (0.34-1.78) | 0.555 | 0.72 (0.31-1.67) | 0.439 | 0.74 (0.31-1.79) | | 0.507 |
| FBG (mmol/L) |  | |  | |  | | |
| Q1 (≤5.13) | Reference | | Reference | | Reference | | |
| Q2 (5.14-5.45) | 0.42 (0.16-1.11) | 0.080 | 0.40 (0.14-1.10) | 0.075 | 0.37 (0.13-1.04) | | 0.060 |
| Q3 (5.46-5.96) | 0.62 (0.25-1.54) | 0.304 | 0.54 (0.22-1.36) | 0.190 | 0.49 (0.19-1.23) | | 0.129 |
| Q4 (>5.96) | 0.68 (0.33-1.40) | 0.290 | 0.57 (0.27-1.19) | 0.136 | 0.50 (0.24-1.05) | | 0.067 |
| PBG (mmol/L) |  | |  | |  | | |
| Q1 (≤5.4) | Reference | | Reference | | Reference | | |
| Q2 (5.5-6.2) | 0.44 (0.15-1.32) | 0.143 | 0.44 (0.15-1.33) | 0.148 | 0.45 (0.15-1.37) | | 0.162 |
| Q3 (6.3-7.5) | 0.84 (0.35-2.00) | 0.695 | 0.79 (0.33-1.89) | 0.593 | 0.69 (0.29-1.67) | | 0.411 |
| Q4 (>7.5) | 0.87 (0.41-1.87) | 0.721 | 0.87 (0.40-1.88) | 0.724 | 0.78 (0.36-1.69) | | 0.523 |
| TyG index |  | |  | |  | | |
| Q1 (≤8.386) | Reference | | Reference | | Reference | | |
| Q2 (8.387-8.753) | 0.78 (0.46-1.34) | 0.375 | 0.70 (0.40-1.22) | 0.208 | 0.65 (0.37-1.13) | | 0.126 |
| Q3 (8.754-9.157) | 1.05 (0.65-1.70) | 0.853 | 0.99 (0.61-1.63) | 0.975 | 0.87 (0.51-1.48) | | 0.597 |
| Q4 (>9.157) | 0.99 (0.59-1.67) | 0.983 | 0.93 (0.55-1.58) | 0.798 | 0.77 (0.42-1.39) | | 0.379 |
| Model 1: adjusted for age and gender.  Model 2: adjusted for variables in model 1, as well as education level, smoking status, drinking status, physical activity intensity, sleep duration, anemia, excessive salt intake and medication history of hypertension, diabetes and hyperlipidemia.  Model 3: adjusted for variables in model 2 plus BMI, MAP, LDL-C, HDL-C and TG.  HbA1c, glycated hemoglobin; Q, quartile; PBG, postprandial blood glucose; FBG, fasting blood glucose; TyG index, triglyceride-glucose index; HR, hazard ratio; CI, confidence interval. | | | | | | | |
